# Combining independent decisions increases diagnostic accuracy of reading lumbosacral radiographs and magnetic resonance imaging

**DOI:** 10.1371/journal.pone.0194128

**Published:** 2018-04-03

**Authors:** Ralf H. J. M. Kurvers, Annemarie de Zoete, Shelby L. Bachman, Paul R. Algra, Raymond Ostelo

**Affiliations:** 1 Center for Adaptive Rationality, Max Planck Institute for Human Development, Lentzeallee, Berlin, Germany; 2 Department of Health Sciences, Amsterdam Public Health research institute, Vrije Universiteit, Amsterdam, the Netherlands; 3 Department of Radiology, Medical Centre Alkmaar, Alkmaar, the Netherlands; Fundacao Champalimaud, PORTUGAL

## Abstract

Diagnosing the causes of low back pain is a challenging task, prone to errors. A novel approach to increase diagnostic accuracy in medical decision making is collective intelligence, which refers to the ability of groups to outperform individual decision makers in solving problems. We investigated whether combining the independent ratings of chiropractors, chiropractic radiologists and medical radiologists can improve diagnostic accuracy when interpreting diagnostic images of the lumbosacral spine. Evaluations were obtained from two previously published studies: study 1 consisted of 13 raters independently rating 300 lumbosacral radiographs; study 2 consisted of 14 raters independently rating 100 lumbosacral magnetic resonance images. In both studies, raters evaluated the presence of “abnormalities”, which are indicators of a serious health risk and warrant immediate further examination. We combined independent decisions of raters using a majority rule which takes as final diagnosis the decision of the majority of the group. We compared the performance of the majority rule to the performance of single raters. Our results show that with increasing group size (i.e., increasing the number of independent decisions) both sensitivity and specificity increased in both data-sets, with groups consistently outperforming single raters. These results were found for radiographs and MR image reading alike. Our findings suggest that combining independent ratings can improve the accuracy of lumbosacral diagnostic image reading.

## Introduction

Low back pain is a major health problem in the industrialized world. In the United States it is, for example, the second most common reason for visiting health care workers, and approximately 1/3 of adults in the US reports low back pain during the last 3 months [1–5]. Accurately diagnosing the causes of low back pain is, however, known to be particularly challenging [6–8]. A widely used method to aid health care providers in diagnosing the causes of low back pain is the use of lumbar and lumbosacral spine imaging (i.e., radiography, computerized tomography (CT) and magnetic resonance (MR) imaging). The use of these techniques has dramatically increased in recent years, despite severe criticism of their validity and effectiveness, and practice guidelines advising against the routine use of such techniques [6,9–11]. Studies evaluating the validity and reliability of different lumbosacral spine image reading methods have reported mixed results, ranging from low to high levels of validity and reliability [12–16]. Taken together, all of this suggests that diagnosing low back pain is a highly complex task. Establishing new means of improving diagnostic accuracy for image reading in the context of low back pain is thus imperative.

One hitherto unexplored mechanism for increasing diagnostic accuracy of spine image reading is to apply a collective intelligence approach. Collective intelligence refers to the phenomenon that multiple minds can solve cognitive tasks better than single minds [17–22]. Collective intelligence can arise via different mechanisms, such as group discussions with consensus seeking (e.g., Delphi-technique, nominal group technique) but also by algorithmically combining independent decisions. Here we will focus on the latter approach. Previous work has shown the potential of such an approach in increasing the diagnostic accuracy of dermatologists evaluating skin lesions [23,24], radiologists evaluating mammograms [24,25], clinicians predicting positive bone scans [26], and medical students diagnosing simulated patients arriving at the emergency room [27]. However, the extent to which collective intelligence could improve diagnostic accuracy in the case of difficult-to-diagnose low back pain is currently unknown.

In this study, we investigated the benefits of utilizing a collective intelligence approach as a means of increasing diagnostic accuracy of interpreting lumbosacral radiographs and MR images. The objective was to compare the performance of the majority rule (a powerful collective intelligence rule which combines the independent decisions of multiple raters) against the performance of single raters, both in terms of sensitivity and specificity. We used two different individual benchmarks: the performance of the average single individual in a group, and the performance of the best single individual in a group.

## Materials and methods

**Fig 1:**
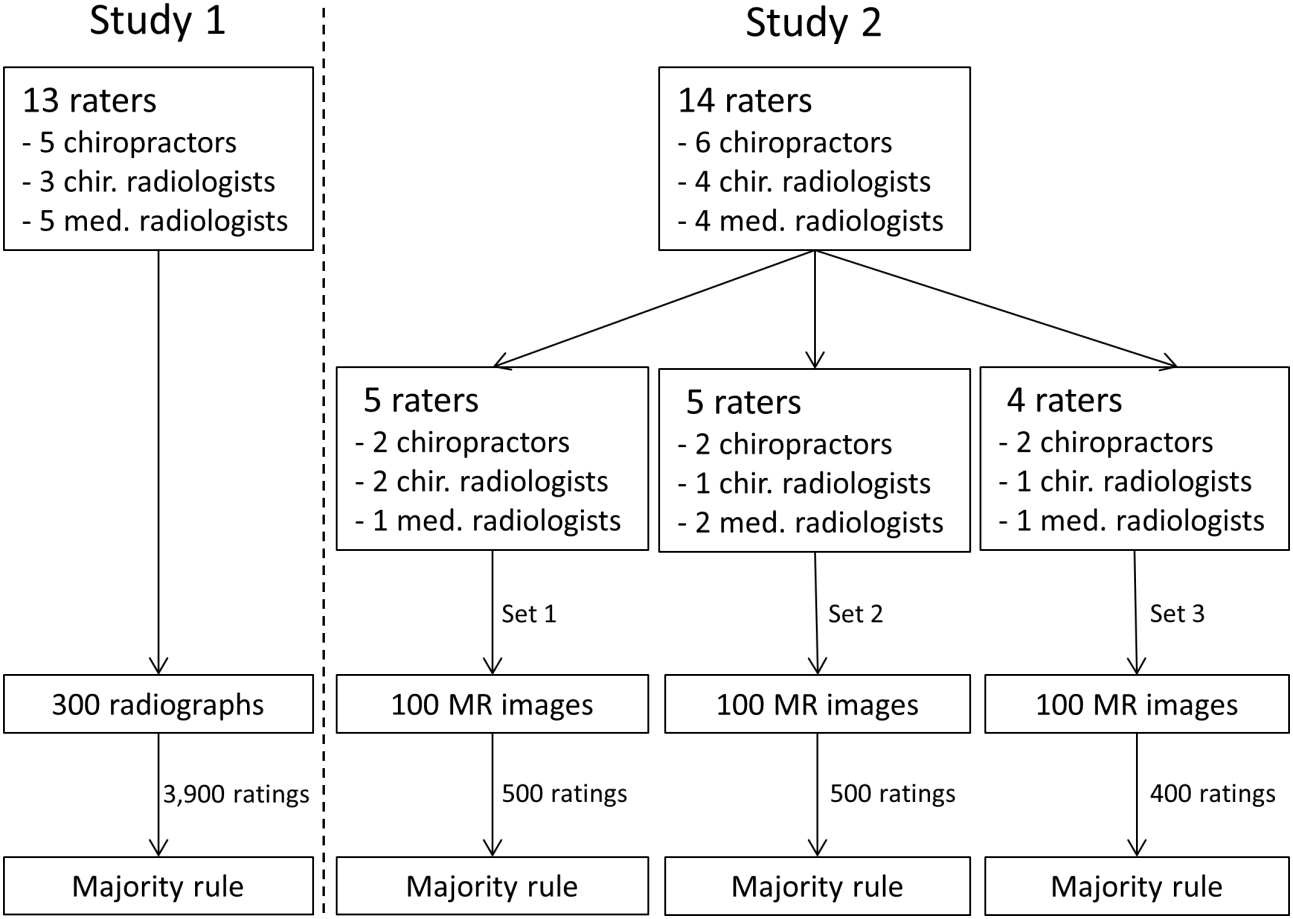
Flow diagram showing the procedures of both studies. In study 1, all 13 raters evaluated all 300 radiographs. In study 2, five raters evaluated 100 MR images belonging to set 1; five raters evaluated 100 MR images belonging to set 2; and four raters evaluated 100 MR images belonging to set 3. Chir. radiologists = Chiropractic radiologists. Med. radiologists = Medical radiologists.

We used two data-sets from two previously-published studies ([15,16] see also Fig 1 and S1 File). We briefly describe both studies below and refer for further methodological details to the original studies due to space constraints.

### Study 1 radiographs

Study 1 investigated the reliability of lumbosacral spine radiograph reading by chiropractors (n = 5), chiropractic radiologists (n = 3) and medical radiologists (n = 5) (for rater details see [15]). Each of the 13 raters independently rated a set of 300 patient-blinded lumbosacral radiographs. Radiographs were derived from a database from the Medical Centrum Alkmaar (the Netherlands). Only radiographs that involved the entire lumbar vertebrae and more than half of the sacrum and from patients greater than or equal to 18 years of age were included (for detailed description of image selection see [15]). The study was designed to investigate the reliability of detecting “significant abnormalities”, defined as conditions which have a major influence on the continued well-being of a patient. When detected, these conditions warrant immediate referral to a hospital or the intervention needs to be modified. The significant abnormalities included: infection (n = 7), malignancy (n = 15), fracture (n = 8), inflammatory spondylitis (n = 6) and spondylolysis-spondylolisthesis (n = 14). 50 out of 300 radiographs used in the study thus had a significant abnormality (i.e., prevalence: 16.7%). The reference test was based on clinical findings, if present lab data, and/or MR/CT imaging. A chiropractor and medical expert in spinal radiology checked that the abnormality was detectable and of sufficient quality.

All of the 13 raters were fully licensed from their professional organizations. Each rater independently evaluated the set of 300 radiographs twice, three months apart. For the purpose of this study, we consider only the first session of evaluations. Before the rating started, a meeting was organized to ensure uniform interpretation of the rating list. For each radiograph, a rater indicated whether the radiograph in question contained an “abnormality” or not. Raters were not informed about the total number of abnormalities present. For further experimental details of the study see [15].

### Study 2 MR images

Study 2 investigated the reliability of lumbosacral spine MR image reading by chiropractors (n = 6), chiropractic radiologists (n = 4) and medical radiologists (n = 4) (for full rater details see [16]). Each rater was assigned to one of three image sets, each containing 100 unique, patient-blinded MR images (sets 1 & 2 were evaluated by five raters and set 3 by four raters, respectively).

Three hundred MR images of the lumbosacral spine of patients referred by primary care clinicians and specialists were selected retrospectively from the Medical Centrum Alkmaar (the Netherlands). Only images from patients greater than or equal to 18 years of age and of sufficient image quality were selected (for detailed description of image selection see [16]). The reference test was based on the evaluations by one experienced chiropractic radiologist and one experienced medical radiologist (who were not raters in the actual study). If they disagreed, a third medical radiologist had the final say. All three experts were specialized in musculoskeletal imaging and they had substantially more years of experience (respectively, 8, 19 and 26) than the 14 raters (median number of years’ experience of raters: 6 years). Nonetheless, the use of an expert panel as reference test is not optimal. To investigate this issue further, we studied the number of cases in which the expert panel disagreed on whether the image should be labelled as specific finding or not (see below). The experts disagreed on six images [16]. Therefore, we also analysed our results while excluding these six ambiguous cases. We further revisit this issue in the discussion.

Specific findings were defined as infections, malignancies, fractures, herniated disc and central stenosis. In case of such specific findings, patients need to be referred to a medical specialist or the treatment needs to be modified. Two classifications were used, because the criteria to define nerve root involvement in disc herniation and central stenosis are not always unambiguous and because we used an expert panel as reference test. In classification ‘A’ infection, malignancy, fracture, herniated disc with definitive root involvement and central stenosis with definite nerve root involvement were classified as “specific finding”. In classification ‘B’ herniated disc and central stenosis with doubtful nerve root involvement were also classified as “specific finding”. Each of the three image sets had approximately equal prevalence of specific findings (prevalence classification ‘A’: set 1: 32%, set 2: 30%, set 3: 31%; classification ‘B’: set 1: 57%, set 2: 56%, set 3: 57%). The image sequence was randomized differently for each rater.

Each rater rated each set of MR images on the presence of the five specific findings. These were then dichotomized in “abnormal” and “normal”. As in Study 1, raters evaluated MR images in two sessions. For the purpose of this study, we consider only the first session of evaluations. For further details of the study see [16].

For both studies, the medical ethical committee of the Alkmaar hospital approved the study. Potential participants were approached via an email, containing information about the study. This was followed up by a phone call to the potential participants in which the study was explained in more detail. Participants who then expressed verbal consent (that is, after receiving full information about the study) were enrolled in the study.

In sum, Study 1 comprised 300 lumbosacral spine radiographs all rated by 13 raters (3,900 ratings in total). Study 2 comprised three sets of 100 unique lumbosacral spine MR images, whereby set 1 and 2 were evaluated by five raters, and set 3 by four raters (1,400 ratings in total) (see also Fig 1). This allowed us to test how combining independent ratings affected collective accuracy using a majority rule.

### Majority rule versus the average individual performance

We investigated the performance of the majority rule, which chooses as final diagnosis the diagnosis receiving most support. The majority rule is a powerful method for boosting collective accuracy under a wide range of individual accuracy levels and decision making contexts [28–31]. To test how group size affected collective accuracy, we tested a range of group sizes. For study 1, we tested group sizes ranging from 3 to 9; for study 2, we tested group sizes ranging from 3 to 5. We could not test larger group sizes in Study 2 because raters only evaluated the MR images within their own test set (Fig 1). Furthermore, we only evaluated odd group sizes to avoid the need of a tie-breaker rule. Within each study, and for study 2 within each test set, we randomly drew groups of *n* raters. We then pooled all the ratings of the *n* raters and evaluated for each radiograph (study 1) / MR image (study 2) whether more raters were in favor of ‘normal’ (in which case the diagnostic image was labeled normal) or ‘abnormal’ (in which case the diagnostic image was labelled as abnormal). From this, we calculated the performance of the majority rule in terms of (i) sensitivity (i.e., the percentage of abnormal cases correctly diagnosed as abnormal), (ii) specificity (i.e., the percentage of normal cases correctly diagnosed as normal), and (iii) the Youden’s index (*J)*. The Youden’s index combines sensitivity and specificity in one diagnostic measure [32,33] and is calculated as: *J* = sensitivity + specificity– 1. A perfect test has *J* = 1 (i.e., sensitivity = specificity = 1), whereas a test with no discriminatory power has *J* = 0 (i.e., sensitivity = 1—specificity; that is, the test has equal probability of giving a positive result for both normal and abnormal cases). Next, we calculated for each group the average individual performance using the same three performance measures. We then compared the performance of the majority rule to the average individual performance of the same group (i.e., performance majority rule minus average individual performance). For study 1, we repeated this procedure 250 times for each group size (i.e., 3, 5, 7 and 9). We compared whether the resulting distributions were statistically higher than zero (i.e., the null hypothesis that there is no difference between the performance of the majority rule and the average individual group performance) at the P < 0.05 level. For 250 groups, this implies that the number of groups at or below zero should not exceed ⌊ 250 · 0.05 =⌋ 12. For study 2, we could not perform the same number of simulations because each of the raters evaluated only one out of three image sets. Therefore, we created the maximum number of unique groups possible at each group size, which was 24 for group size 3, and only two for group size 5. To be statistically significant at the P < 0.05 level at group size 3, the number of groups with values at or below zero should not exceed ⌊ 24 · 0.05 =⌋ 1. For group size 5, the number of unique groups was limited to two. Therefore, we were not able to draw any statistical inference for this group size. Additionally, we used a permutation test for paired samples (comparing the performance of the majority rule with the average individual performance of that group) to verify our findings. This was done using the coin package in R (version 3.2.2).

Finally, we tested whether group size affected improvement in the three performance measures (i.e., Youden’s index, sensitivity and specificity) using the full range of group sizes tested. We used general linear models (LMs), using group size (continuous) as a fixed effect and the improvement in the performance measure as response variable, running a different model for each performance measure. This analysis was done in R (version 3.4.0) and significance levels were derived from the t-values and associated p-values.

### Majority rule versus the best individual

Next, we investigated under which conditions combining the decisions of raters allowed the collective to outperform the best individual rater in a group. Thus, in this case we did not take the average individual performance of a group as the benchmark but instead the performance of the best rater in a given group. We specifically investigated how differences in average individual performance among group members affected the ability of a group to outperform the best single rater in that group. This was done because recent work in visual perception tasks found that combining decisions of individuals of similar individual performance level resulted in a collective performance which was better than any single individual [34–37]. However, when individuals substantially differ in their average individual performance level, combining their decisions leads to worse performance level as compared to the performance of the best individual. (Similar results were recently found in breast and skin cancer diagnostics [24].) Since both the similarity between raters in a group and the identity of the best rater in a group need to be known before they can be used (in practice), we performed a cross validation procedure. First, within each study, we created all possible combinations of raters at group size 3. For each group, we randomly assigned 70% of all images to a training set (210 and 70 images for study 1 and 2 respectively) and the remaining 30% to a test set (90 and 30 images for study 1 and 2). The training set was used to (i) determine the identity of the best performing rater of the triplet in terms of the Youden’s index, and (ii) determine the similarity in accuracy between group members. For this, we calculated the Youden’s index for each group member in the training set and used the mean pairwise absolute deviation (MPAD) to calculate the similarity in *J* among group members.

$$\text{MPAD}=\frac{2}{n\left(n-1\right)}*\sum_{i<j}\left|J_{i}-J_{j}\right|,$$

where *n* is the number of raters *i* and *j*. This measure is thus the expected absolute difference in *J* between two randomly chosen group members. Next, we determined the performance of the best individual (selected from the training set) in the test set, as well as the performance of the majority rule in the test set. Performance was again measured in terms of (i) sensitivity, (ii) specificity, and (iii) the Youden’s index. We repeated this procedure 500 times for each unique group composition, and averaged results within unique groups. We then studied how the “similarity in accuracy” (estimated from a training set) affected the performance of the majority rule in the test set as compared to the performance of the best rater (also selected from the training set) in the test set. For this, we analyzed the effect of “similarity in accuracy” on a group’s ability to outperform its best individual using LMs in R. Significance levels were derived from the t-values and associated p-values.

## Results

### Majority rule versus the average individual performance

**Fig 2:**
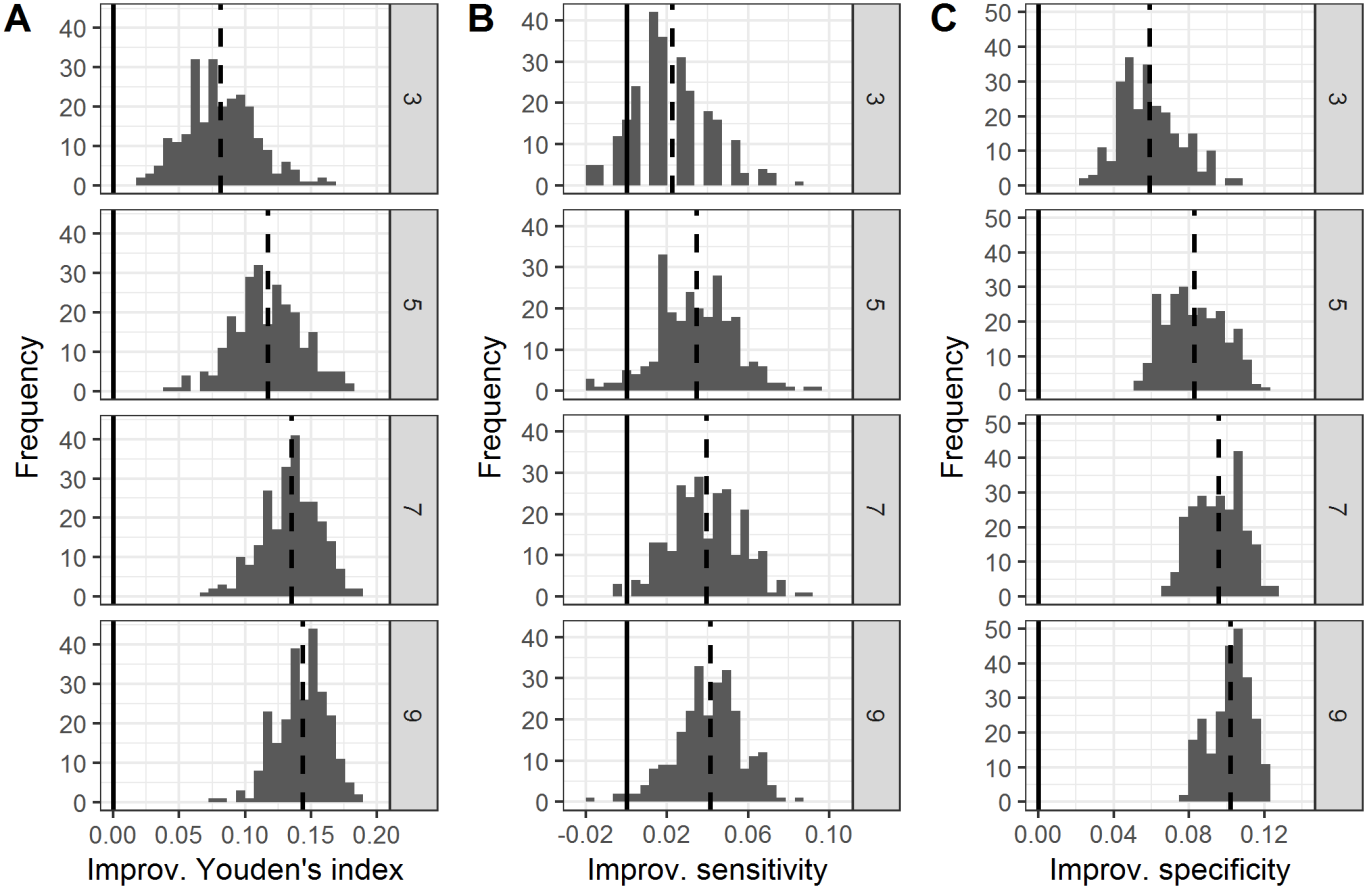
Effect of group size on the Youden’s index, sensitivity and specificity for reading lumbosacral spine radiographs (study 1). Histograms show the frequency distributions of the improvement of groups under the majority rule as compared to the average individual performance of that group, in terms of (A) the Youden’s index, (B) sensitivity, and (C) specificity. At each group size (numbers in grey panels), 250 unique groups were drawn. Values higher than zero indicate that the majority rule was better than the average individual performance of that group. Negative values indicate that the majority rule was worse than the average individual performance of that group. The dashed vertical lines show the mean value of each distribution. The solid vertical lines represent the average individual group performance (which by definition corresponds to an improvement of zero). Improv = Improvement.

Fig 2 shows the performance of the majority rule compared to the average individual performance of that group for study 1 (Radiographs). At each group size, 250 simulations were performed. For all group sizes, the majority performance in terms of the Youden’s index of all the 250 simulated groups was better than the average individual performance of that group (i.e., none of the distributions overlapped with zero) (Fig 2A). This provides statistical evidence in favor of a significant difference from zero at all group sizes. For sensitivity, the 95% CIs at group sizes 3 and 5 overlapped with zero and were thus not significantly different from zero (Fig 2B). However, for group sizes 7 and 9, the distributions were significantly greater than zero (Fig 2B). For specificity, none of the distributions overlapped with zero (Fig 2C). The permutation tests for paired samples largely confirmed these findings: all P < 0.01 for each combination of group size (3, 5, 7 and 9) and performance measure (Youden’s index, sensitivity and specificity).

**Fig 3:**
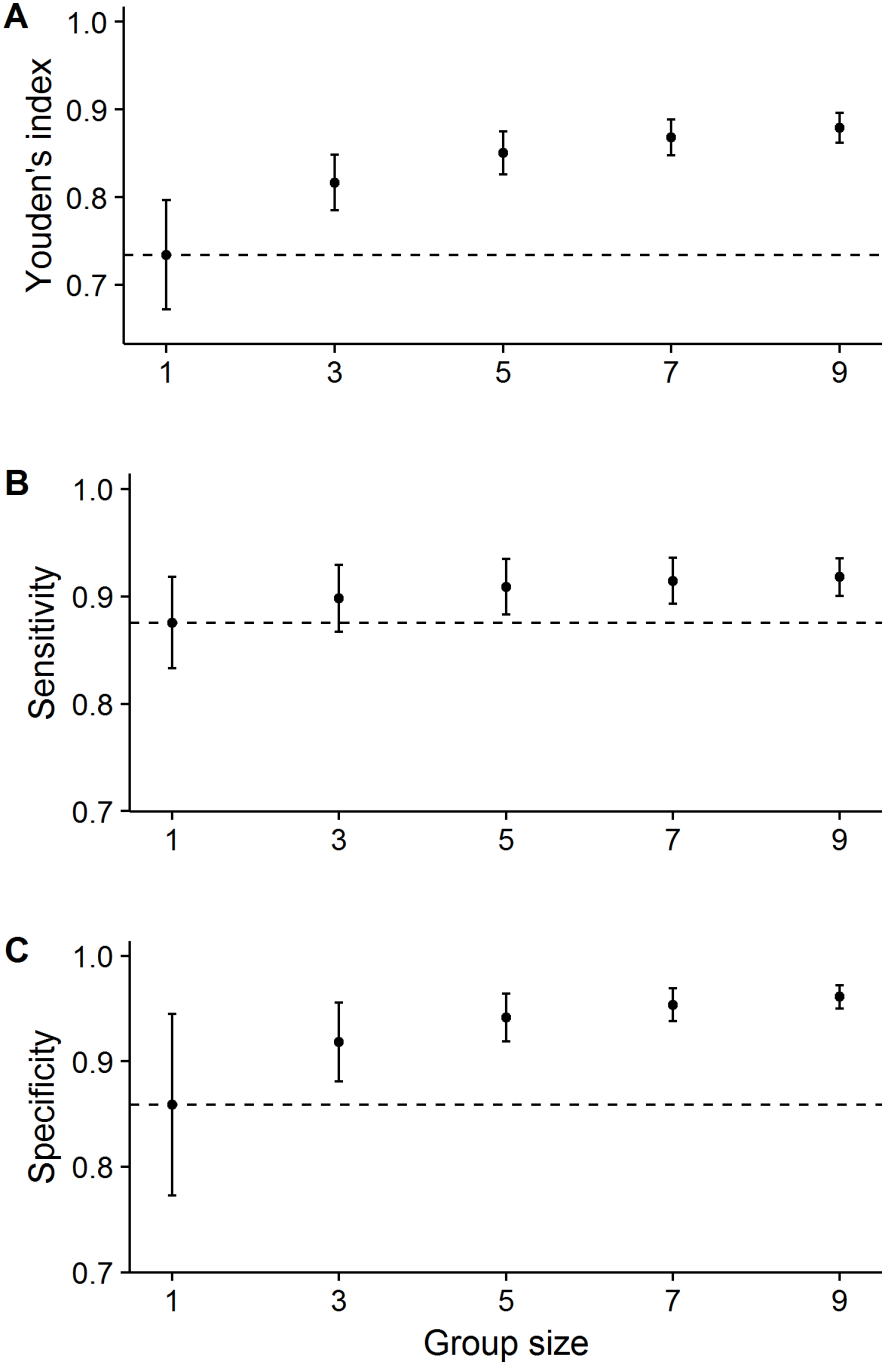
Effect of group size on the absolute performance in terms of the Youden’s index, sensitivity and specificity for reading lumbosacral spine radiographs (study 1). Increasing the number of independent ratings under the majority rule increased (A) the Youden’s index, (B) sensitivity and (C) specificity. Horizontal lines show the average individual performance (i.e., group size = 1). Error bars represent standard deviation.

Fig 3 shows the absolute performance of the different group sizes, illustrating how all three performance measures (Youden’s index, sensitivity and specificity) increased with increasing group size (results of LMs using full range of group sizes: all P < 0.01). The largest improvements in all measures arose when the number of raters increased from one to three, illustrating that the largest gains were obtained at the lower end of the group size range.

**Fig 4:**
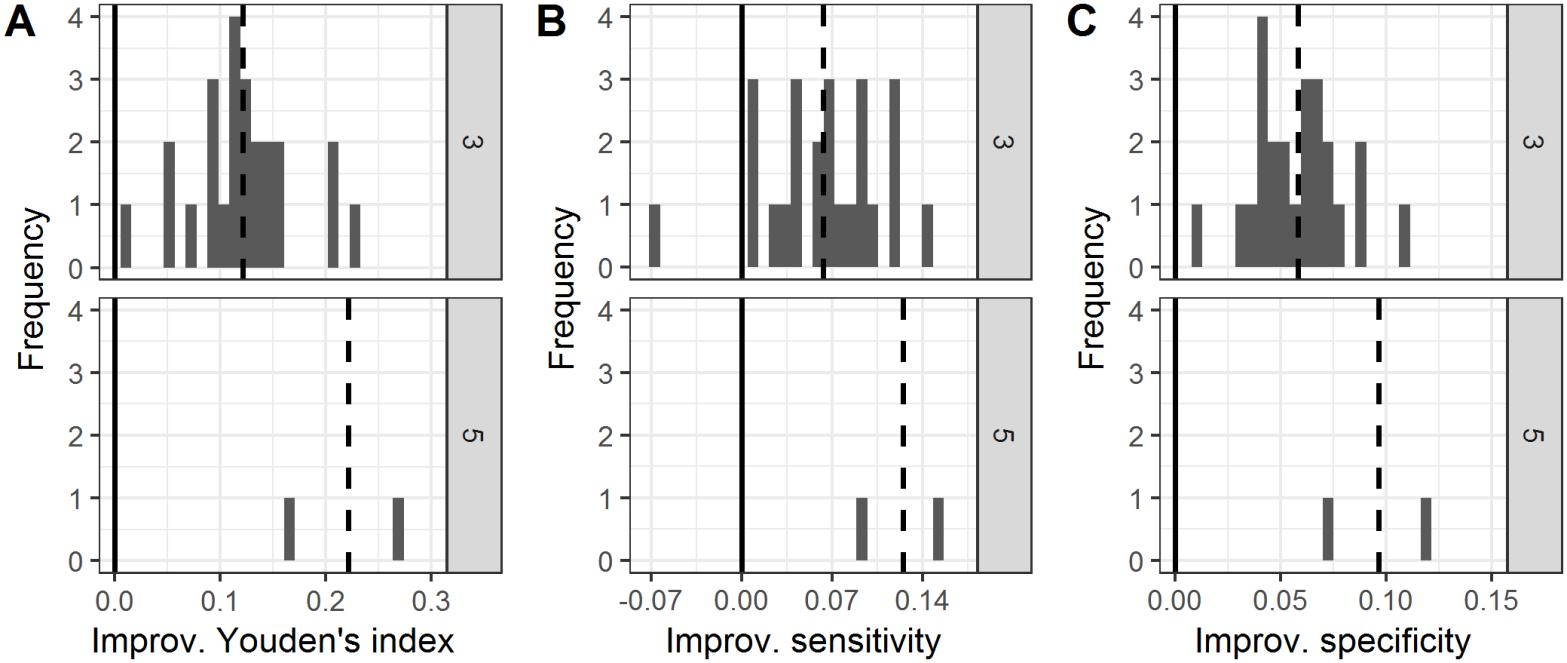
Effect of group size on the Youden’s index, sensitivity and specificity for reading lumbosacral spine MR images (study 2). Histograms show the frequency distributions of the improvement of groups under the majority rule as compared to the average individual performance of that group, in terms of (A) the Youden’s index, (B) sensitivity, and (C) specificity. At group size three, 24 unique groups were available, and at group size five, two unique groups. Values higher than zero indicate that the majority rule was better than the average individual performance of that group. Negative values indicate that the majority rule was worse than the average individual performance of that group. The dashed vertical lines show the mean value of each distribution. The solid vertical lines represent the average individual group performance (which by definition corresponds to an improvement of zero). Improv = Improvement.

Study 2 consisted of three test sets (each containing 100 unique MR images), rated by respectively five, five and four raters. Within each test set, we evaluated the performance of the majority rule using classification A. Fig 4 shows the performance of the majority rule compared to the average individual performance of that group, for group sizes 3 (24 unique groups) and 5 (only 2 unique groups). At group size 3, the majority performance in terms of the Youden’s index of all the 24 simulated groups was better than the average individual performance of that group (i.e., the distribution did not overlap with zero) (Fig 4A). Furthermore, the improvements in sensitivity (Fig 4B) and specificity (Fig 4C) at group size 3 were also significantly greater than zero. The permutation tests for paired samples confirmed these findings: P < 0.01 for all three performance measures (Youden’s index, sensitivity and specificity).

**Fig 5:**
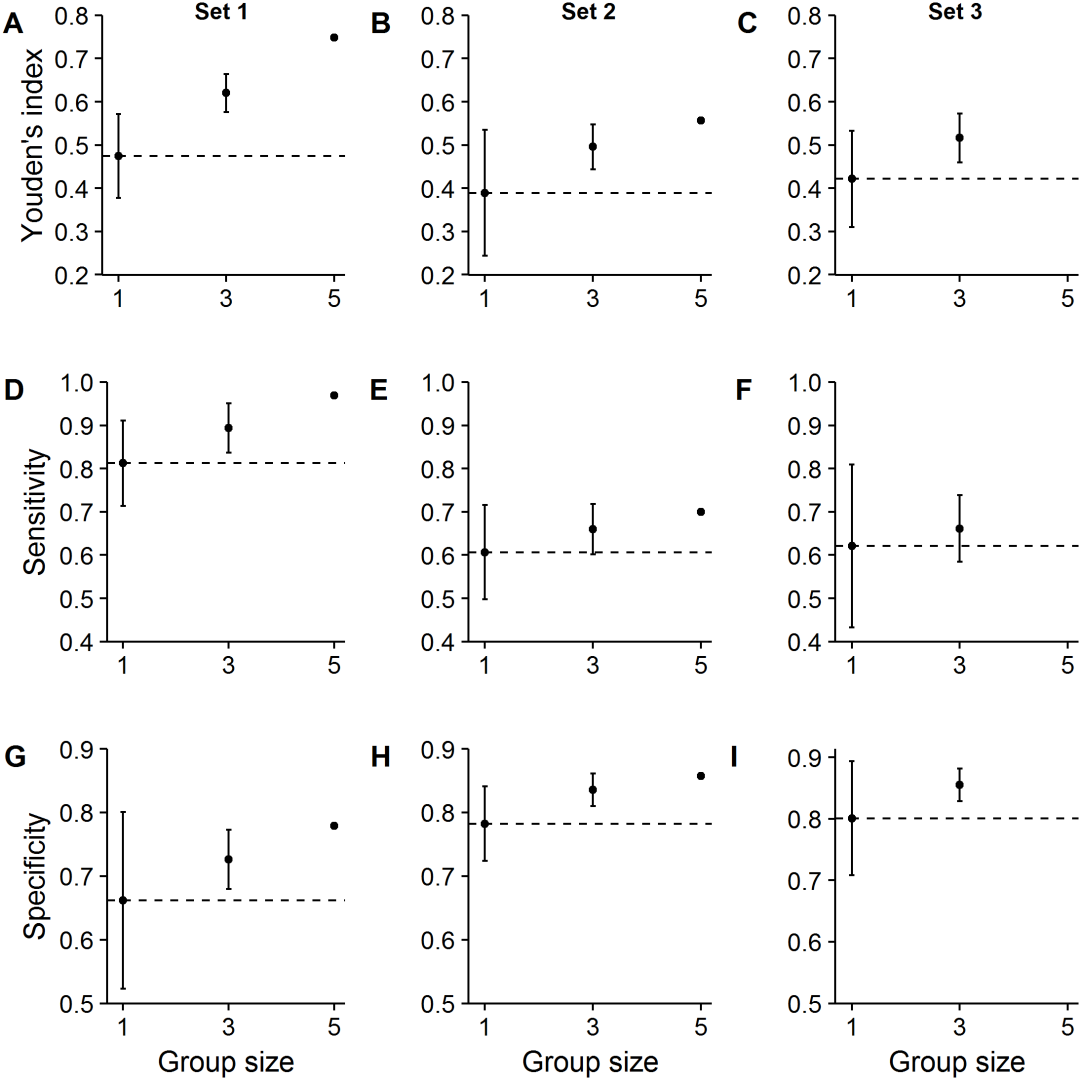
Effect of group size on the absolute performance in terms of the Youden’s index, sensitivity and specificity for reading lumbosacral spine MR images (study 2). Increasing the number of independent ratings under the majority rule increased (A-C) the Youden’s index, (D-F) sensitivity and (G-I) specificity in all three test sets. Horizontal lines show the average individual performance (i.e., group size = 1). Error bars represent standard deviation (S.D.). Each test set contained 100 unique MR images rated by respectively five, five and four raters, explaining why group size five in test set 3 is absent, and the absence of S.D. for group size five in test set 1 and 2 (since there was only one unique combination of five raters).

Fig 5 shows the absolute performance of the different group sizes, for each of the three image sets. For all sets, applying the majority rule consistently increased the Youden’s index (Fig 5A–5C), sensitivity (Fig 5D–5F) and specificity (Fig 5G–5I) (results of LMs: all P < 0.01). Despite substantial differences in average individual sensitivity/specificity between the three test sets, we found that in all tests increasing group size consistently increased both sensitivity and specificity. We found similar results when looking at ratings using classification B (S1 Fig). Finally, we also found similar results (for classifications A and B) when excluding the six cases for which the expert panel could not find consensus (S2 Fig).

### Majority rule versus the best individual

**Fig 6:**
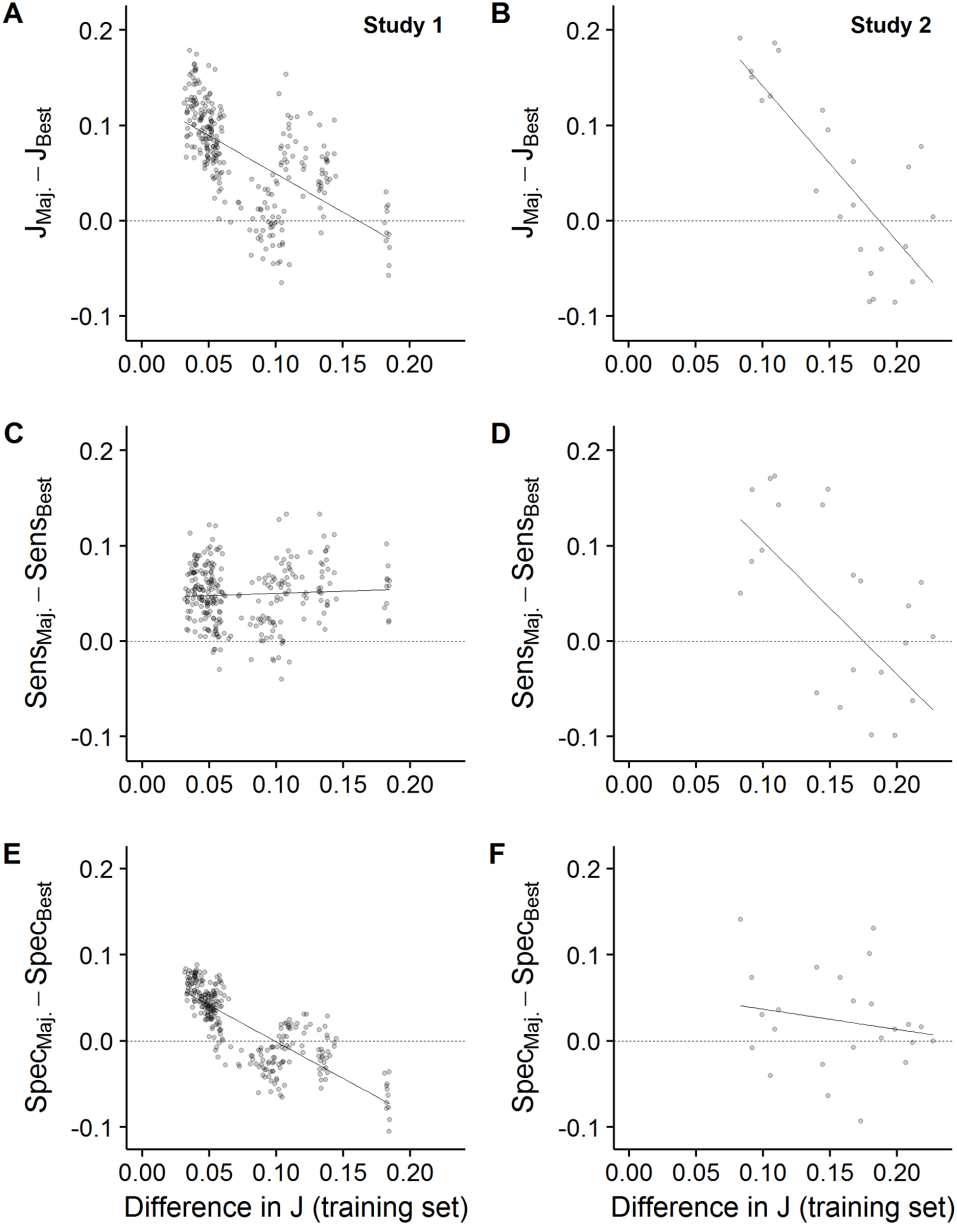
Performance difference between the majority rule and the best rater in a group of three raters as a function of the difference in performance level (i.e., difference in Youden’s index) between raters. Each point represents a unique combination of three raters. Values above zero indicate that combining independent ratings under the majority rule outperformed the best rater in that group by that many percentage points in terms of (A, B) the Youden’s index (*J*) (C, D) sensitivity (sens) and (E, F) specificity (spec). Values below zero indicate that the best rater outperformed the majority rule. Lines are linear regression lines. Data are shown separately for (A, C, E) Study 1 and (B, D, F) Study 2. Results show groups averages based on a cross validation procedure with 500 repetitions per unique group composition (see Methods and materials for more details).

In both studies, we found that as the difference in performance levels among the three raters (in the training set) increased, the performance of the majority rule (in the set) as compared to the best rater (in the test set) decreased (Youden’s index: results of LM, Study 1: estimate (est) ± se = -0.80 ± 0.06, t = -12.81, p < 0.001, Fig 6A; Study 2: est ± se = -1.63 ± 0.27, t = -5.97, p < 0.001, Fig 6B). When raters had relatively similar individual performance level (i.e., ΔJ < 0.17), combining their decisions under the majority rule led to better decisions as compared to the best single rater (Fig 6A and 6B). In contrast, when raters were relatively dissimilar in individual performance level (ΔJ > 0.17), combining their decisions led to worse decisions as compared to the best single rater. When separating the overall performance (i.e., Youden’s index) into sensitivity and specificity (Fig 6C–6F), we observe that the negative relationship between performance difference and the ability of a group to outperform the best rater, is driven by specificity in Study 1 (Fig 6E) but by sensitivity in Study 2 (Fig 6D). (LM, Study 1: sensitivity: est ± se = 0.05 ± 0.05, t = 0.98, p > 0.3; specificity: est ± se = -0.85 ± 0.04, t = -22.3, p < 0.001; Study 2: sensitivity: est ± se = -1.39 ± 0.43, t = -3.20, p = 0.004; specificity: est ± se = -0.23 ± 0.27, t = -0.88, p > 0.3).

## Discussion

This study showed that pooling independent ratings increases the diagnostic accuracy in lumbosacral spine image interpretation: increasing the number of independent ratings increased both sensitivity and specificity. These results were found in both studies, each one using a different imaging technique (i.e., radiographs and MR images respectively). Our results corroborate earlier findings in different domains of medical diagnostics which have shown an increased diagnostic accuracy when pooling independent diagnostic decisions in radiology [24,25], dermatology [23,38] positive bone scan predictions [26] and emergency medicine [27].

Although groups in general outperformed the average performance of single raters, whether groups also outperformed the best rater in a group depended critically on the similarity in performance level among raters (Fig 6A and 6B). When raters were of similar performance, combining their ratings resulted in outcomes which were better than those of any of the group members. However, when group members differed too much in their individual performance level, combining their decisions resulted in worse outcomes as compared to the best rater. In practice, this implies that if there is no prior information on average individual performance levels, then combining ratings/decisions is to be preferred (as combining outperforms the average performance). However, whenever prior information on rater’s performance is available, then this could be used to determine whether combining should be preferred over the best individual, or to combine specific raters of similar individual performance. Our separate treatments of sensitivity and specificity (Fig 6C–6F) show that considering these two key dimensions of performance can be important when doing this, since these performance measures might scale differently with rater similarity.

Another method for combining the decisions of multiple raters is to conduct a group discussion followed by a joint group decision. Group discussions have been shown to increase performance in several domains [39–42], but at the same time several studies have highlighted the pitfalls associated with group discussions, including social loafing, group think and obedience to authority [43–45]. We currently do not know how our mechanism of combining independent decisions compares to scenarios with group discussions followed by a joint group decision, and future research could pitch these collective mechanisms against each other to compare the potential collective gains of both methods [46]. Future studies investigating combining independent ratings in low back diagnoses could also collect data on the confidence raters have in their decision which would allow testing other, more complex, collective intelligence rules such as (weighted) confidence rules [24,34,35,47] which give more weight to highly confident decisions. Future studies would also benefit from a higher number of independent raters. In our simulations, we created unique groups, but these groups consisted (partly) of the same raters, introducing dependence between groups. In an ideal scenario, more raters would be available to avoid such dependencies. Simultaneously, obtaining a large sample of medical experts can be a challenge so such ideal scenarios have to be traded off against practical feasibility.

The costs and benefits of using lumbosacral spine imaging for patients with low back pain is heavily debated and current guidelines advise against the use of routine imaging. Only in the presence of progressive neurological deficits or symptoms suggesting a serious or specific underlying condition, is the use of imaging recommended [3,7]. Our study did not directly address the reliability and validity of imaging under different patient specific scenarios. Our study does, however, show that when imaging is used, there is scope for improving the diagnostic accuracy by combining independent decisions of raters. Importantly, this improvement arose in the two key dimensions of diagnostic accuracy: sensitivity and specificity. Improvements in one dimension thus did not go at the expense of improvements on the other dimension, as for example was the case in some studies investigating the diagnostic accuracy of second opinions in mammography. Several of these studies reported that second opinions increased sensitivity (as compared to single decision-makers) but at the expense of decreased specificity [48–50].

One important limitation of our study constitutes the reference tests used. Although Study 1 used a combination of clinical findings, if present lab data, MR/CT imaging, and an expert panel, the reference test in study 2 was solely based on a consensus expert panel. An important assumption we made is thus that the ratings of the expert panel correlate with the truth. The gold standard for identifying serious underlying pathologies in low back pain remains a challenge, as there is a lack of a gold reference standard. In the literature, there is a large variation of reference tests. To illustrate, in a Cochrane review on the diagnostic accuracy of MRI on low back pain, studies were only included if they used surgery, expert panel consensus or diagnostic work up as reference standard. Surgery, especially when combined with clinical follow-up, is often regarded as the best reference test, but subject to partial verification as often only patients with a strong suspicion of a specific underlying cause will be subjected to surgery. Verification bias might lead to a higher sensitivity and a lower specificity but it has also been found that it increases both sensitivity and specificity [51]. More studies are needed to identify best possible imaging strategies in patients with chronic low-back pain, symptoms of radiculopathy or spinal stenosis, patients assessed in referral settings, and other specific subgroups [6]. Another limitation of our study is that in both data-sets we analyzed, the prevalence of “abnormalities” was substantially higher than in clinical practice, implying that the collective gains we found cannot be directly transferred to clinical populations. Future studies using more realistic prevalence rates could investigate the consequences for clinical populations directly. Despite the elevated prevalence rates, it is noteworthy that in all the data-sets we investigated, combining decisions improved sensitivity and specificity; despite there being substantial differences in average individual sensitivity and specificity levels (e.g., Fig 5). This suggests that our findings are, at least partly, independent of the exact average sensitivity and specificity level of raters. A further important consideration of our study is that applying a collective intelligence approach requires more viewing time by health care providers. These additional costs need to be weighed against the potential gains: increased sensitivity (i.e., higher detection rates of serious abnormalities) and increased specificity (potentially lowering costly unnecessary or even harmful follow-up treatments). Future studies could quantify how to optimize benefits of a collective intelligence approach while balancing costs.

## Conclusions

Our findings suggest that employing a collective intelligence approach can improve both sensitivity and specificity of lumbosacral diagnostic imaging reading. These results were found in two different studies using different imaging methods (i.e., radiographs and MR images).

## Supporting information

S1 Fig Effect of group size on the Youden’s index, sensitivity and specificity for reading lumbosacral spine MR images (study 2) using classification B. Histograms show the frequency distributions of the improvement of groups under the majority rule as compared to the average individual performance of that group, in terms of (A) the Youden’s index, (B) sensitivity, and (C) specificity. At group size three, 24 unique groups were available, and at group size five, two unique groups. Values higher than zero indicate that the majority rule was better than the average individual performance of that group. Negative values indicate that the majority rule was worse than the average individual performance of that group. The dashed vertical lines show the mean value of each distribution. The solid vertical lines represent the average individual group performance (which by definition corresponds to an improvement of zero). Improv = Improvement. At group size three, the majority performance was significantly better than the average individual performance in terms of the Youden’s index and sensitivity, but not in terms of specificity.
(TIFF)

S2 Fig Effect of group size on the Youden’s index, sensitivity and specificity for reading lumbosacral spine MR images (study 2) using classification A and B when excluding the six ambiguous cases for which the expert panel could not find consensus. Histograms show the frequency distributions of the improvement of groups under the majority rule as compared to the average individual performance of that group, in terms of (A, D) the Youden’s index, (B, E) sensitivity, and (C, F) specificity. At group size three, 24 unique groups were available, and at group size five, two unique groups. Values higher than zero indicate that the majority rule was better than the average individual performance of that group. Negative values indicate that the majority rule was worse than the average individual performance of that group. The dashed vertical lines show the mean value of each distribution. The solid vertical lines represent the average individual group performance (which by definition corresponds to an improvement of zero). Improv = Improvement. At group size three, the majority performance was significantly better than the average individual performance in all six panels, except for specificity under classification B.
(TIFF)

S1 File Data sets Kurvers et al. 2018. The two data sets used in this study.
(XLSX)
