# Supplementary figures and images for: Combining independent decisions increases diagnostic accuracy of reading lumbosacral radiographs and magnetic resonance imaging
Source: PLoS One. 2018 Apr 3;13(4):e0194128. doi: 10.1371/journal.pone.0194128 (PMC5882099; doi:10.1371/journal.pone.0194128)

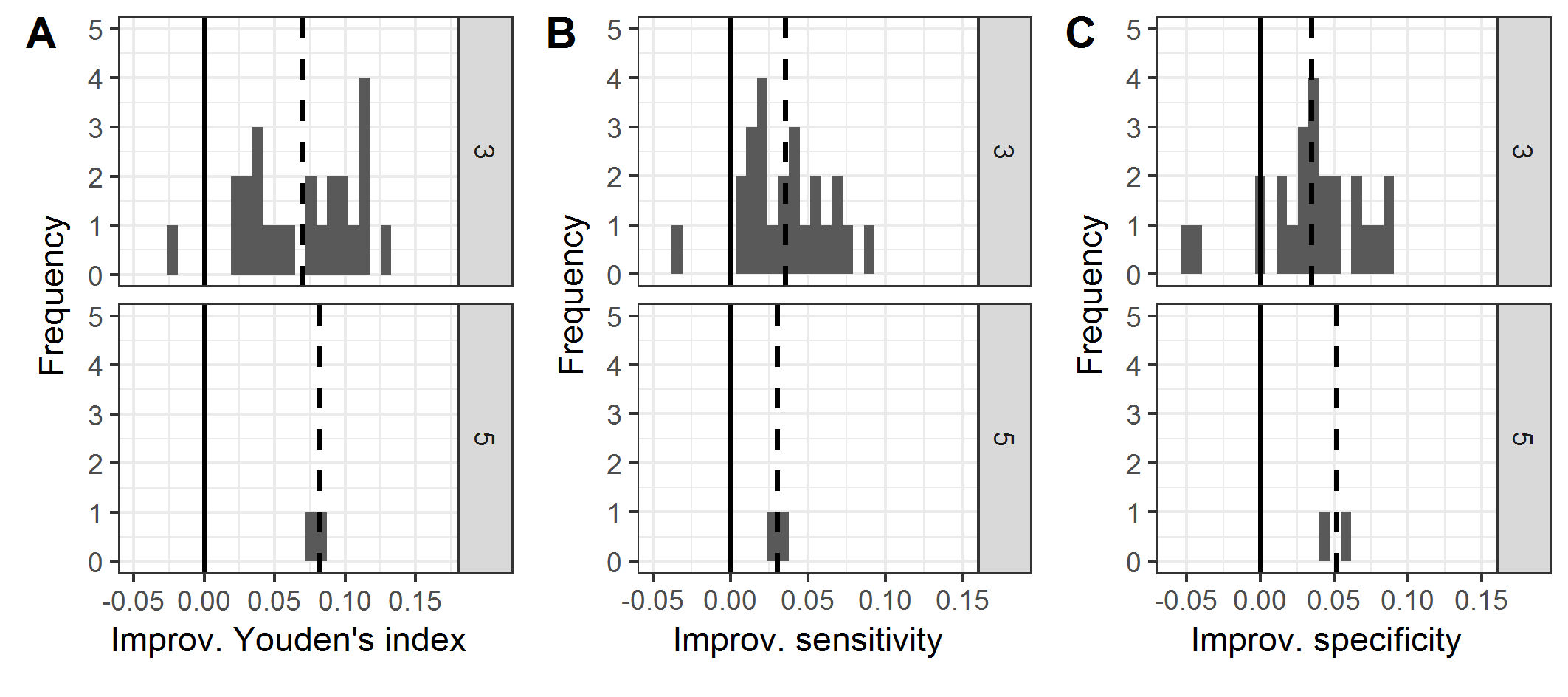

Supplement: S1 Fig — Histograms show the frequency distributions of the improvement of groups under the majority rule as compared to the average individual performance of that group, in terms of (A) the Youden’s index, (B) sensitivity, and (C) specificity. At group size three, 24 unique groups were available, and at group size five, two unique groups. Values higher than zero indicate that the majority rule was better than the average individual performance of that group. Negative values indicate that the majority rule was worse than the average individual performance of that group. The dashed vertical lines show the mean value of each distribution. The solid vertical lines represent the average individual group performance (which by definition corresponds to an improvement of zero). Improv = Improvement. At group size three, the majority performance was significantly better than the average individual performance in terms of the Youden’s index and sensitivity, but not in terms of specificity. (TIFF) [file pone.0194128.s001.tiff]

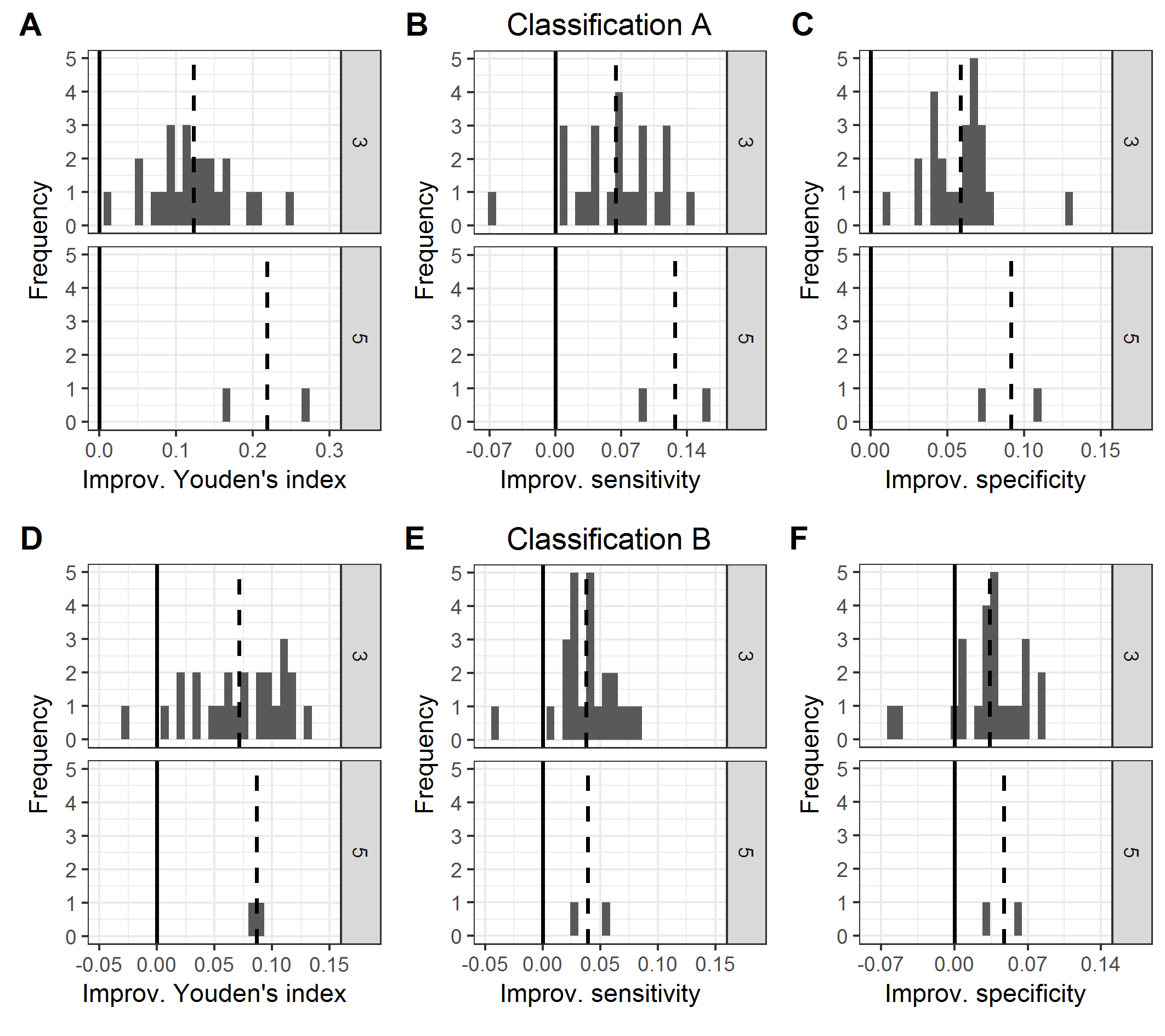

Supplement: S2 Fig — Histograms show the frequency distributions of the improvement of groups under the majority rule as compared to the average individual performance of that group, in terms of (A, D) the Youden’s index, (B, E) sensitivity, and (C, F) specificity. At group size three, 24 unique groups were available, and at group size five, two unique groups. Values higher than zero indicate that the majority rule was better than the average individual performance of that group. Negative values indicate that the majority rule was worse than the average individual performance of that group. The dashed vertical lines show the mean value of each distribution. The solid vertical lines represent the average individual group performance (which by definition corresponds to an improvement of zero). Improv = Improvement. At group size three, the majority performance was significantly better than the average individual performance in all six panels, except for specificity under classification B. (TIFF) [file pone.0194128.s002.tiff]
